# Dickkopf-1 Is Oncogenic and Involved in Invasive Growth in Non Small Cell Lung Cancer 

**DOI:** 10.1371/journal.pone.0084944

**Published:** 2013-12-31

**Authors:** Shujun Li, Xuebo Qin, Xin Guo, Airong Cui, Yuzheng He, Sen Wei, Xiaolu Wang, Baoen Shan

**Affiliations:** 1 The Second Hospital of Hebei Medical University, Shijiazhuang, China; 2 Hebei Chest Hospital, Shijiazhuang, China; 3 Tianjin Lung Cancer Institute, Tianjin Medical University General Hospital, Tianjin, China; 4 The Fourth Hospital of Hebei Medical University, Shijiazhuang, China; Virginia Commonwealth University, United States of America

## Abstract

Dickkopf-1 (DKK1) is an inhibitor of the Wnt/β-catenin signaling pathway. However, the role of DKK1 in the progression of non small cell lung cancer (NSCLC) is not fully understood. In this study, RT-PCR and Western blot were used to examine the expression of DKK1 in a panel of ten human NSCLC cell lines and NSCLC tissues. DKK1 expression was highly transactivated in the great majority of these cancer lines. The expression of DKK1 was upregulated on both mRNA and protein levels in NSCLC tissues compared with the adjacent normal lung tissues. Immunohistochemistry and immunofluoresence revealed that DKK1 was mainly distributed in the cytoplasm in both carcinoma tissues and cell lines. DKK1 protein expression was also evaluated in paraffin sections from 102 patients with NSCLC by immunohistochemistry, and 65(63.73%)tumors were DKK1 positive. Relative analysis showed a significant relationship between DKK1 positive expression and lymph node metastasis(*P*<0.05). Patients with DKK1-positive tumors had poorer DFS than those with negative ESCC (5-year DFS; 15.4% versus 27%, P = 0.007). To further explore the biological effects of DKK1 in NSCLC cells, we over-expressed DKK1 in NSCLC 95C cell using eukaryotic expression vector pCMV-Tab-2b and performed a knockdown of DKK1 in LTEP-a-2 cell using a short hairpin RNA expression vector pSilencer 5.1. DKK1 did not have any effect on proliferation, but seemed to play a role in migration and invasion capability. Overexpression of DKK1 promotes migratory and invasive activity of 95C, while DKK1 knockdown resulted in the suppression of migration and invasion potentials of LTEP-a-2 cell. Taken together, these results indicate that DKK1 may be a crucial regulator in the progression of NSCLC. DKK1 might be a potential therapeutic target in NSCLC.

## Introduction

Non small cell lung cancer (NSCLC) is one of the most common malignancies and the incidence is increasing[1-4]. Despite the advances in early detection and improvements in the treatment, long-term survival of NSCLC remains unsatisfactory. Tumor relapse and metastasis are the main influence factors of prognosis. Biomarkers that can predict the risk of recurrence and the metastasis are extremely urgent. Therefore, it is necessary to identify novel targets that participate in the tumor progression and design appropriate treatment strategies for NSCLC patients.

Dickkopf-1 (DKK1) is a secreted protein involved in Wnt signaling pathway acting as an inhibitor. In the conventional Wnt/β-catenin, Wnt-1 protein binds to the frizzled receptor (Fz) and the low-density lipoprotein receptor-related protein-5/6 (LRP5/6), triggering signals for proliferation via β-catenin [5,6]. DKK1 binds to LRP5/6 and blocks interaction with Wnt-1, resulting in β-catenin degradation and retardation of proliferation [7-10]. The expression and roles of DKK1 is different in various cancers, current studies have reported that overexpression of DKK1 is found in many malignant tumors including breast cancer, lung cancer, esophageal carcinomas and hepatocellular carcinoma (HCC)[11-15], indicating a potential oncogenic function of DKK1[16]. In spite of these studies, there little has been reported on the significance of DKK1 expression in NSCLC progression and prognosis.

In this study, we first analyzed the expression of a panel of human NSCLC cell lines and 102 resected specimens of NSCLC, and explored the correlation between DKK1 expression and clinicapathological factors. Subsequently we detected the biological effects of DKK1 on migration and invasion in cultured pancreatic carcinoma cells.

## Materials and Methods

### Ethics Statement

This study was approved by the ethical committees of the Second Hospital of Hebei Medical University and Tianjin Chest Hospital.All the participants provided their written informed consent to participate in this study.

### Cell cultured and transfection

The lung andenocarcinoma cell line A549 and lung large cell line NCI-H460 were purchased from ATCC. The lung andenocarcinoma cell lines SPC-A-1, LTEP-a-2, GLC82 A2 and PC-9; lung squamous cell lines YTMLC-9 and lung large cell lines 95C, 95D are gifted from Tianjin lung cancer institute [14]. The cells were cultured in RPMI-1640 (Invitrogen, Carlsbad, CA) medium containing 10% fetal bovine serum (FBS, GIBCO), 100 IU/ml penicillin and 100mg/ml streptomycin maintained at 37°C in humidified air containing 5% CO_2_. 

 The cells were cultured to 80% confluence and transfected with recombined eukaryotic vector and empty vector using Lipofectamine 2000 (Invitrogen, CA, USA) according to the manufacturer’s recommendation.

### Patient samples

In this study, 102 patients’ formalin-fixed NSCLC tissue samples were used for immunohistochemistry staining which were obtained from the Second Hospital of Hebei Medical University. Ten patient fresh tissues including primary NSCLC and matched adjacent normal tissues were obtained from Tianjin Chest Hospital. All the tissues were stored in liquid nitrogen prior to use. Tissue samples were grinded in liquid nitrogen to isolate total RNA and protein. 

### Plasmid construction

 The eukaryotic expression vector pCMV-Tag-2b (Invetrogen) was reconstructed to express DKK1. 815 base pairs long DKK1 sequence (NM: 012242.2) including *EcoR I* and *BamH I* restriction enzyme sites was amplified from genomic DNA. The primer sequences were: Forward 5’-TGGATCCATGATGGCTCTGGGCGCAGCGGGAG-3’, Reverse:5’-CGAATTCTTAGTGTCTCTGACAAGTGTGAAGCCTAGAAG-3’. The PCR amplified product was subcloned into pCMV-Tag-2b vector. The recombinant vector was designated as pCMV-Tag-2b-DKK1. The knockdown vector was constructed by using a eukaryotic expression vector pSilencer 5.1 (Ambion). The target sequence of DKK1 gene was 5’-AATAAGTACCAGACCATTGAC-3’, and the resultant vector was designated as pSilencer-DKK1. The negative control sequence was 5’-CTACCGTTGTTATAGGTG-3’. The negative control siRNA plasmid (pSilencer-NC) encodes a siRNA, which has no significant sequence similarity to human gene sequences. All the construction sequences were designed according to Ambion’s online siRNA Target Finder. Sequence analysis was conducted to verify the resulted vectors. 

### Western blotting

 Western blot was performed to detect the expression of DKK1 in resected NSCLC specimens and NSCLC cell lines. Frozen NSCLC specimens were grinded in liquid nitrogen; cell lines were cultured to 80% confluence and harvested. Tissue samples and cells were lysed in RIPA lysis buffer (phosphate-buffered saline containing 1% Triton X-100 and 1nM PMSF) at 4°C for 30 min and centrifuged at 12,000rpm for 15min. The protein concentration was quantified using the BCA protein assay (Pierce, Rockford, IL). Equal quantities of protein were loaded and electrophoreses in a 10% SDS-PAGE gel, which was then transferred to nitrocellulose (NC) membrane. The membrane was incubated for 60 min in PBS containing 0.1% Tween-20 and 5% skimmed milk to block any nonspecific binding; this was followed by incubation at 4°C with anti-human DKK1 rabbit polyclonal antibody (LifeSpan, WA, USA, 1:500 dilutions). The membrane was washed three times for 10 min in PBS with 0.1% Tween-20 and then incubated for 1 h with HRP-conjugated bovine anti-rabbit (1:5000 dilutions) secondary antibody (Boster Biological Technology, Wuhan, China) at room temperature. The immumoreactive proteins were then detected using ECL substrate following manufacturer’s recommendation. β-actin was used as an endogenous protein for normalization. IPP image analysis software was used for quantification. 

### RT-PCR and real-time PCR

 For analysis of DKK1 expression in NSCLC cells, total RNA was isolated with Trizol. Equal mRNA from each sample was reversely transcribed to cDNA using random primer. GAPDH was used as internal control, PCR reactions were carried out by using the following primers: DKK1, Forward 5’-CAACGCTATCAAGAACCTGC-3’, Reverse 5’-GATCTTGGACCAGAAGTGTC-3’; GAPDH, Forward 5’-GGCATGGACTGTGGTCATGA-3’, Reverse 5’-TGGXGTGTGAACCACGAGAA-3’. PCR was optimized for the number of cycles to ensure product intensity to be within the linear phase of amplification, the PCR products were then analyzed by electrophoresis in 2% agarose gel.

Real-time PCR was carried out using SYBR**^®^** Green (Code DRR041A, Takara) in a total volume of 30μl over a two-step cycles using the following temperature protocol: 10 sec at 95 °C followed by 42 cycles of 95 °C for 15 s and 55 °C for 30s. The reactions were placed in a 96-well plate (ABI) using a preheated real-time instrument (ABI 7500HT). The relative levels of expression were quantified and analyzed using Bio-Rad iCycler iQ software. Three independent experiments were performed to analyze relative gene expressions and each sample was tested in triplicate. Ct values were used to calculate the expression of mRNA levels. The amount of target gene expression (2-Ct) was normalized using the endogenous GAPDH reference, the amount of target gene in the control sample was set as the calibrator at 1.0. Primer sequences used for real-time PCR were listed in Table S1.

### Proliferation assay

 MTT assay was used to analyze cell proliferation. After 24h of transfection, cells were seeded into 96-well plate at 5.0×10^3^ cells/ml and cultured for 24 h, 48 h, 72h, and 96h respectively. At each time point, 10μl MTT reagent (5mg/ml, Sigma) was added to each well, and incubated for 4h at 37°C. 200μl DMSO (Invitrogen) was added to dissolve the formazan crystals for 30 min after discarding the supernatant. Spectrometric absorbance was measured at a wavelength of 490nm on microplate reader (Spectra Max M5, MD, USA). To ensure the results each sample was tested in triplicate and all the experiments were performed three times. 

### Wound healing assay

The migration ability was determined using wound-healing assay. Equivalent cells were plated into 12-well plates without antibiotics. After 24h of transfection, cells were wounded with a sterile plastic 100μl micropipette tip, and the floating debris were washed with PBS and cultured in serum-free medium. Width of the wound was measured at different time points. 3-4 different locations were visualized and photographed under a phase-contrast inverted microscope (40× objective, TE2000-E, Nikon, Japan).

### Boyden chamber assay

Boyden chamber assay was used to examine cell invasion capability. Cells were transfected with lipofectmine 2000. After 16 hours, cells were trypsinized and resuspended. 5.0×10^4^ cells in 300μl RPMI-1640 medium were placed into the upper compartment (pore size 8-μm; BD Biosciences). The lower chambers were filled with 500μl complete medium with 10% FBS. After incubation for 48h at 37°C, the cells on the upper surface of the filter were removed with a cotton swab. The migratory cells on the lower surface of the inserts were fixed and stained with 0.1% crystal violet for 30min at 37°C and washed twice with PBS. The stained cells were then visualized under a microscope and cell number was counted in five random fields (magnifications 100×). Boyden chamber were conducted in duplicate in two separate experiments.

### Immunohistochemistry and immunofluoresence

To investigate the status of the DKK1 protein in clinical NSCLC samples that had been embedded in paraffin blocks, IHC staining was performed according to the following procedure. Paraffin sections were deparaffinized with xylene and rehydrated in graded ethanol solutions. Activity of endogenous peroxidase was blocked with 3% H_2_O_2_ for 15 min at room temperature and then the sections were heated with 0.01M citrate (pH=6.0) at 95°C for 15min in microwave for antigen retrieval. After incubation with anti-DKK1 rabbit polyclonal antibody (ab22827, Abcam Inc, Cambridge, MA, dilution 1:200) for 2h at room temperature, negative controls without the primary antibody. The sections were incubated with HRP-labeled anti-rabbit IgG secondary antibody. The intensity of DKK1 staining was evaluated by using the following criteria [12,14]: strong positive (2+), dark brown staining >50% of tumor cells in cytoplasm; weak positive (1+), any lesser degree of brown staining in tumor cells; absent (scored as 0), immunoreactive to DKK1 10% or less of the tumor cells. The scores were assessed by two pathologists without knowing the clinicopathological data.

TE13 cells were cultured on glass coverslips, which were then fixed with 4% paraformaldehyde and permeabilized with 0.2% Triton X-100 in PBS for 10min at room temperature. Cells were subsequently blocked with 3% BSA for 30min at room temperature, and then incubated with primary antibodies diluted in PBS containing 3% BSA for 60min at room temperature. After being washed with PBS, the cells were stained with FITC-conjugated secondary antibody (Santa Cruz Biotechnology) for 30 min at 37°C. Finally, the coverslips were washed with PBS and nuclei were mounted with DAPI and visualized with confocal laser scanning microscope.

### Statistical Analysis

Statistical analysis was performed with the SPSS 10.0 software. Data are presented as the mean±SD. Student’s t-test and one-way analysis of variance were used. Relationships between grouped variables were analysed using chi-square test. Survival curves were produced according to the method described by Kaplan and Meier. Differences between curves were estimated using log-rank tests. P values <0.05 were considered to be significant. All experiments were performed at least in triplicates [15].

## Results

### DKK1 expression in cultured human NSCLC cell lines

**Figure 1:**
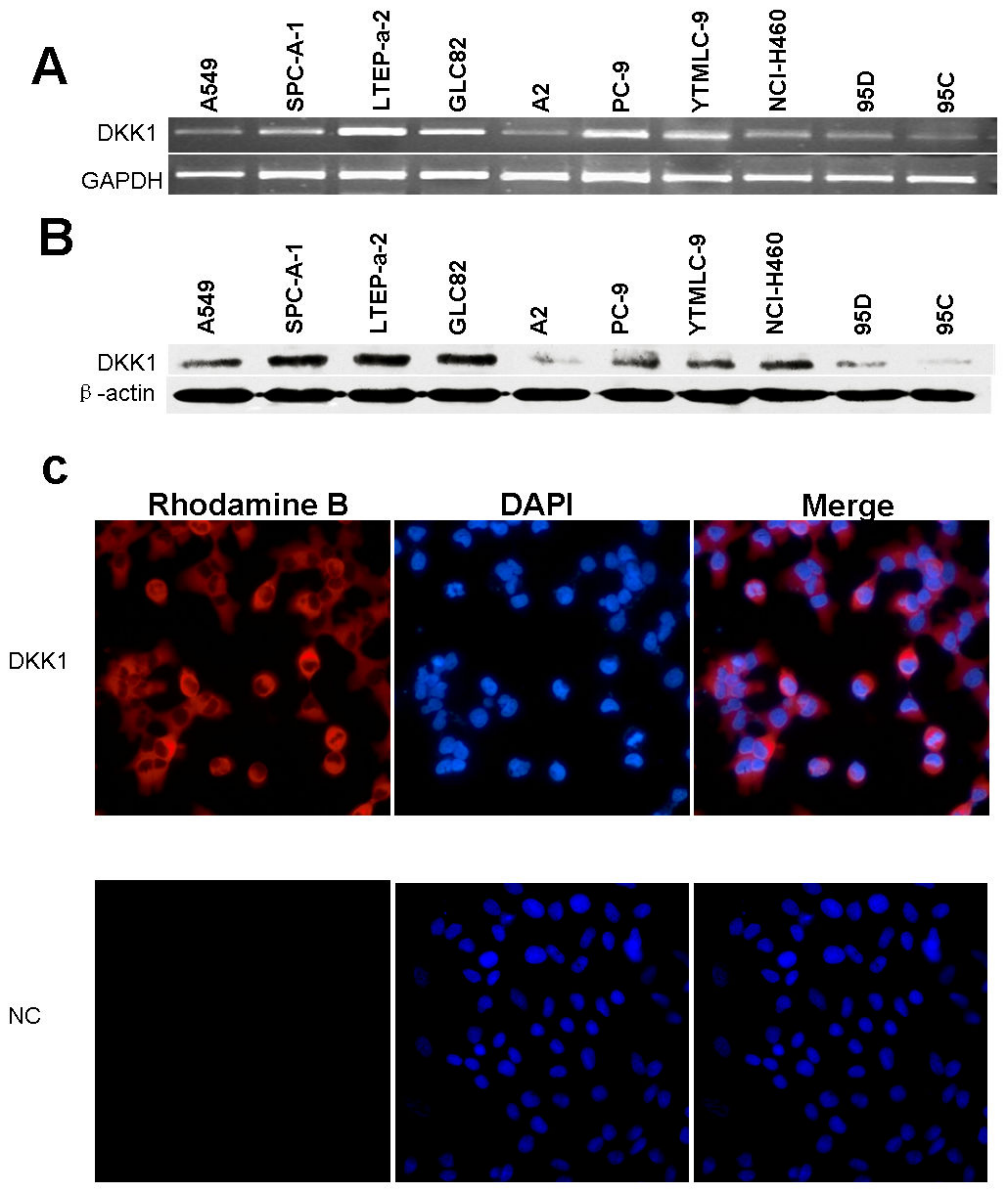
Expression of DKK1 in human NSCLC cell lines. (A) Expression of DKK1 on mRNA level (RT-PCR: 28 cycles of amplification). GAPDH mRNA was used as the internal control. (B) Expression of DKK1 on protein level. β-actin was used as the internal control. (C) Subcellular localization of endogenous DKK1 protein in NSCLC cell. DKK1, stained with rhodamine B, was at the cytoplasm of the cell appearing fine-grained material. Cell nucleus appeared as blue fluorescence stained with DAPI (magnification 100×).

 The expression of DKK1 was characterized in a number of human NSCLC cell lines. DKK1 protein was detectable in all of the cell lines (Figure 1). Higher expression was observed in LTEP-a-2 and GLC-82 cell lines. Lower expression was observed in A2 and 95C cancer cell lines. DKK1 mRNA expression was also examined by using RT-PCR, the results were consistent with the results of Western blot (Figure 1. B). The subcellular localization of DKK1 expression in the NSCLC cell line was defined by immunofluorescent staining. The results showed that DKK1 expression was mostly distributed in the cytoplasm with a granular appearance (Figure 1. C). 

### DKK1 Expression in NSCLC tissues

**Figure 2:**
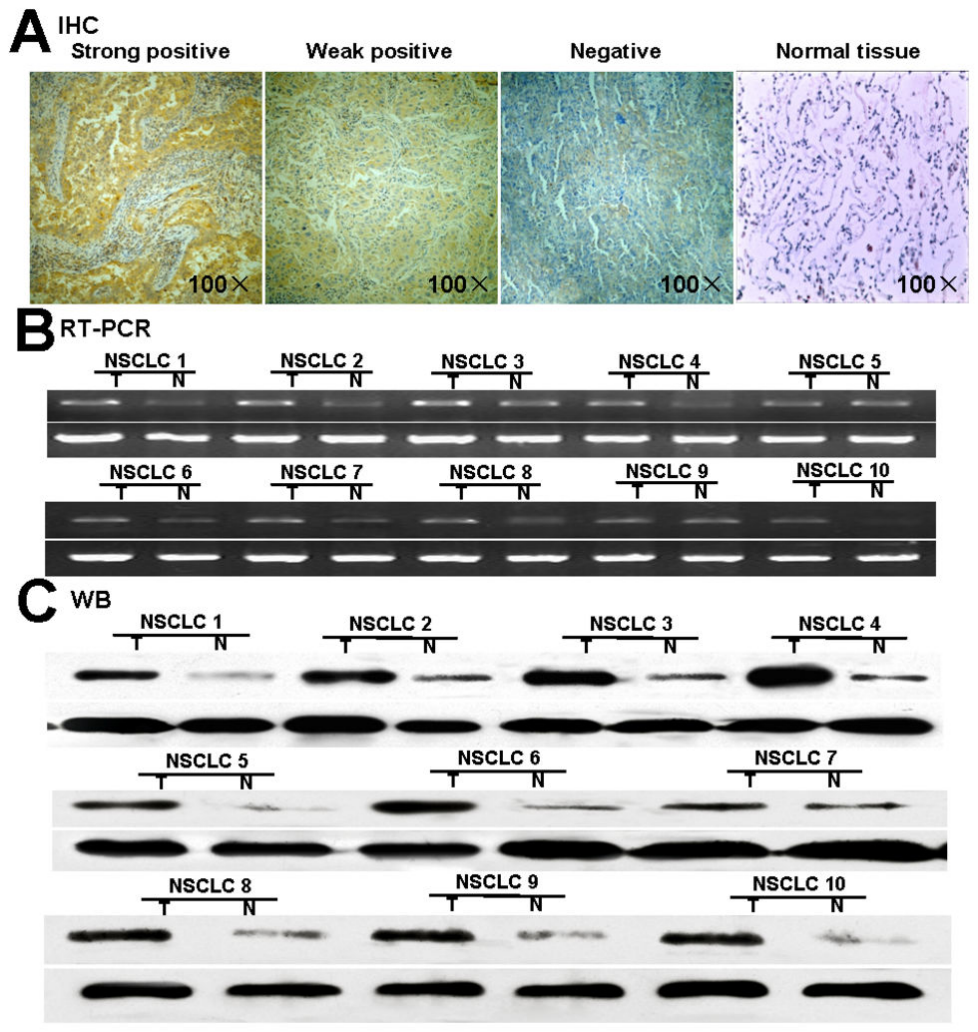
Expression of DKK1 in NSCLC tissues. (A) Expression by immunohistochemical staining. DKK1 strong positive expression in lung cancer showing staining mainly in the cytoplasm of tumor Cells (magnification 100×). Representative DKK1 weak positive lung cancer with pale yellow particles in tumor cells (magnification 100×). DKK1 negative lung cancer cell showing almost no appreciable staining of tumor cells (magnification 100×). Normal lung tissue without staining. (B) Expression of DKK1 mRNA in NSCLC tissues and matched normal lung tissues. (C) Expression of DKK1 protein in NSCLC tissues and matched normal lung tissues. N, Normal tissue; T, Tumor tissue. Positive expressions of DKK1 were mainly accompanied with lymph nodes metastasis.

To examine the expression of DKK1 protein in NSCLC, 102 NSCLC patients paraffin sections were assessed by immunohistochemical analysis. Of these, 46 (45.1%) showed strong positive DKK1 expression, mainly in the cytoplasm of tumor cells, with dark brown granular distribution. 19(18.63%) samples were weak positive expression with pale yellow particles. while the remaining 37(36.27%) were negative. The total rate of positive staining was 63.73%. In contrast, none of the normal epithelium showed significant level of immunohistochemical staining (Figure 2A). We further analyzed the correlation between DKK1 expression and clinicopathological parameters; it is interesting to note that the positive expressions of Dkk1 were mainly accompanied with lymph nodes metastasis (Table 1). The total 5-year disease-free survival (5-year DFS) rate of patients with DKK1-negative was 27%, while patients with DKK1-positive tumors showed poorer DFS than those with negative tumors (5-year DFS; 15.4% versus 27%, P = 0.007) (Figure S1). These results indicate that Dkk1 overexpression is a frequent event in human NSCLC, which might have a potential relationship to the metastasis of NSCLC. 

**Table 1:** Correlation between DKK1 and various clinicopathological parameters ^*^Well group vs. moderate; ^**^T3 vs. T4.

**Parameters**	**n**	**DKK1 expression**		**χ^2^**	***P* value**
		**Positive(%)**	**Negative(%)**		
**Age(years)**					
**<60**	64	39(60.9%)	25(39.1%)	0.578	0.2940
**≥60**	38	26(68.4%)	12(31.6%)		
**Gender**					
**Male**	81	51(63.0%)	30(37.0%)	0.099	0.4820
**Female**	21	14(66.7%)	7(33.3%)		
**Differentiation**					
**Well**	32	21(65.6%)	11(34.4%)	0.064	0.8003^*^
**Moderate**	43	27(62.8%)	16(37.2%)		
**Poor**	27	17(63.0%)	10(37.0%)		
**T**					
**T1**	11	7(63.6%)	4(36.4%)	0.1213	0.7276^**^
**T2**	36	23(63.9%)	13(36.1%)		
**T3**	18	24(66.7%)	12(33.3%)		
**T4**	19	11(57.9%)	8(42.1%)		
**N(Metastasis)**					
**Positive(N1+N2)**	54	47(87.0%)	7(13.0%)	26.976	0.0000
**Negative(N0)**	48	18(37.5%)	30(62.5%)		

 We next observed the expression of DKK1 in surgically resected NSCLC tissues and matched normal tissues. Among 10 cases of NSCLC, 7 showed significantly upregulated expression of DKK1 mRNA in cancer tissue compared with corresponding normal tissue (Figure 2B). Western blot results showed the same tendency on DKK1 protein level (Figure 2C).

### Effect of DKK1 overexpression on migration and invasion in 95C

**Figure 3:**
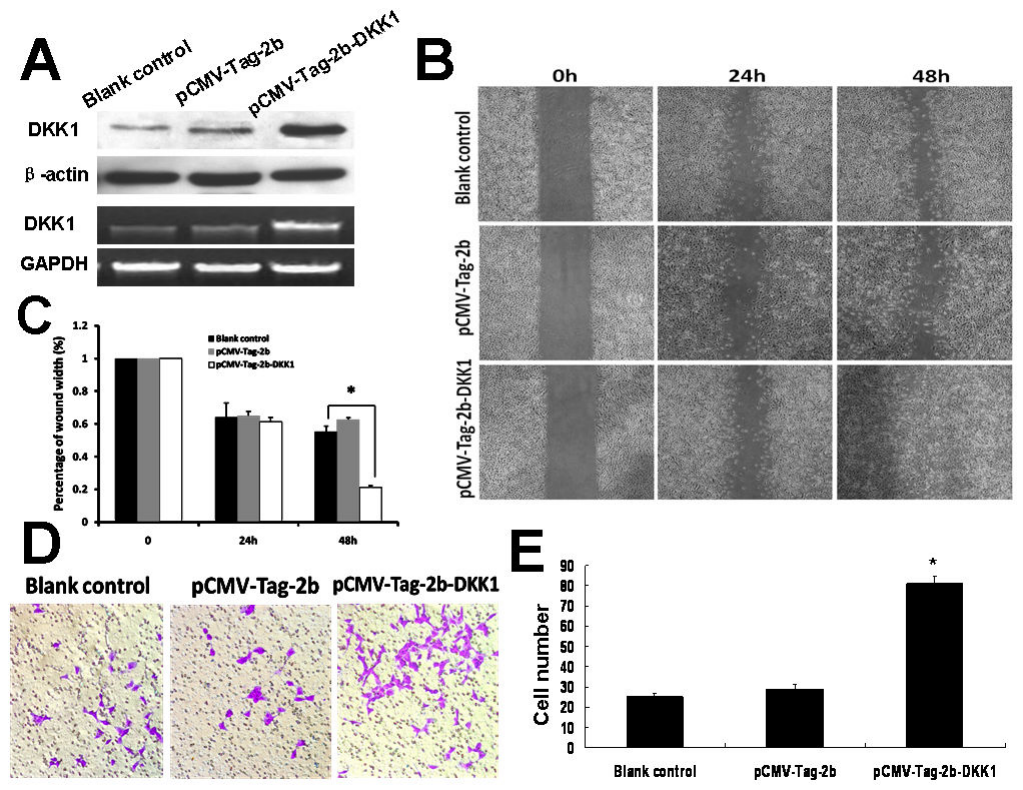
Effects of DKK1 overexpression in 95C cell. (A) RT-PCR and Western blot analysis of the DKK1 expression. DKK1 level in 95C cell transfected with pCMV-Tag-2b-DKK1 is significantly higher than that in pCMV-Tag-2b and blank control group. (B) The wounded and healing 95C cells. (C) Measurement of migration distance (*P<0.05). (D) The invasion ability of 95C transfected with pCMV-Tag-2b-DKK1 was significantly enhanced. (E) The number of cells migrating through the Matrigel-coated filters was statistic analyzed. Assays were done in triplicate wells (*P<0.01).

To test the biological role of DKK1 in NSCLC cell, we examined the consequence of overexpression of DKK1 on cell migration and invasion capability in 95C cell line which actually have a very low level of endogenous DKK1. Eukaryotic expression vector pCMV-Tag-2b-DKK1 was constructed to overexpress the gene DKK1. A significant increase in the levels of both DKK1 protein and mRNA was observed in 95C cell line transfected with pCMV-Tag-2b-DKK1 compared with blank control and negative control (Figure 3. A). Subsequently, MTT assay revealed that overexpression of DKK1 did not alter cell proliferation ability (data not shown). However, the migration ability was promoted in the wound healing assay (Figure 3. B, C). Boyden chamber was used to test the invasiveness, 95C cell were transfected with either pCMV-Tag-2b-DKK1 or pCMV-Tag-2b, and blank control group was added transfection reagent without plasmid. After 18h transfection, the cells were reseeded on top of the insert. Cells that invaded through the barrier and reached the other side of the chamber insert were recorded after 48h of incubation. Boyden chamber results showed that the cells that passed through the membrane in the pCMV-Tag-2b-DKK1 group was greater than the other two groups (Figure 3. D). These observations indicated that overexpression of DKK1 can significantly promote invasion capability of 95C cells (Figure 3. E). 

### Knockdown of endogenous DKK1 by siRNA decreased migration and invasion in LTEP-a-2

**Figure 4:**
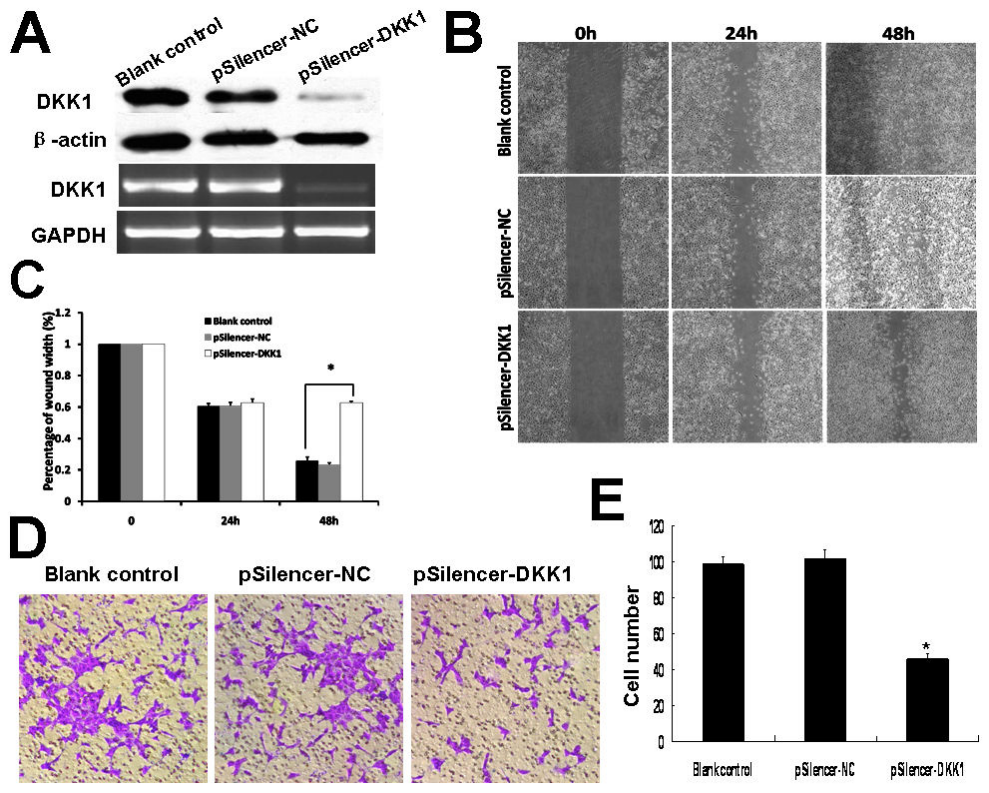
Effects of DKK1 knockdown in LTEP-a-2 cell. (A) RT-PCR and Western blot analysis of the DKK1 expression. DKK1 level in LTEP-a-2 cell transfected with pSilencer-DKK1 is significantly lower than that in pSilencer-NC and blank control group. (B) The wounded and healing LTEP-a-2 cells. (C) Measurement of migration distance (*P<0.05). (D) The invasion ability of LTEP-a-2 transfected with pSilencer-DKK1 was significantly repressed. (E) The number of cells migrating through the Matrigel-coated filters was statistic analyzed. Assays were done in triplicate wells (*P<0.01).

We analyzed the effects of DKK1 knockdown on migration and invasion in LTEP-a-2 cell line. To determine the knockdown efficacy of the shRNA, the DKK1expression level in LTEP-a-2 cell lines was examined by both RT-PCR and Western blot. The specific siRNA effectively reduced the endogenous expression of DKK1 in the cells that were transfected with pSilencer-DKK1 compared with the cells that were transfected with pSilencer-NC vector (Figure 4. A).

Knockdown of endogenous DKK1 by siRNA did not affect LTEP-a-2 proliferation (data not shown), but significantly repressed the migration ability in wound healing assay. The migration rate of pSilencer-DKK1 cells in wound healing was significantly lower after 48h transfection than that of pSilencer-NC and blank control group (Figure 4. B, C). When cultured in Boyden chamber, the number of cells that migrated through the Matrigel-coated porous filters was significantly reduced in DKK1 knockdown pSilencer-DKK1 cells compared with pSilencer-NC and blank control group (Figure 4. D, E).

### Overexpression of DKK1 altered associated genes expression

To explore the mechanism of DKK1 in lung cancer progression, real-time PCR was performed to detect the effect of DKK1 overexpression on the expression of relative genes involved in carcinogenesis. Overexpression of DKK1 did not alter the expression of cell cycle related protein cyclinD1 and apoptosis related proteins Bcl-2 and Box. Akt-1 associated with signaling pathway was minimally upregulated in the 95C cell transfected with Pcmv-Tag2b-DKK1. The expression of MMP2 and VEGFC in the 95C cell transfected with Pcmv-Tag2b-DKK1 was increased 7.78-fold and 2.13-fold compared with control 95C cells respectively (Table S2).

## Discussion

The family of human DKK proteins is composed of DKK1, DKK-2, DKK-3, DKK-4 and a unique DKK3-related protein, termed Soggy[17]. DKK1, encoding a secreted protein, is an antagonist of the Wnt/β-catenin signaling that is involved in tumor progression [13,18-22]. DKK1 is expressed in numerous human cancers; it might play diverse biological roles in tumor cells depending on the cell type involved. DKK1 is upregulated in some kinds of human tumors including NSCLC, hepatocellular carcinoma, pancreatic cancer [12,14,19,23]. It seemed that DKK1 might play crucial roles during the progression of these types of tumors, but the biological effects of DKK1 in NSCLC have not been clarified. In this study, we confirmed that DKK1 is expressed in a panel of NSCLC cell lines. Immunoflouresence showed that DKK1 subcellular location expression was mostly distributed in the cytoplasm of NSCLC cells. The IHC study of DKK1 expression in 102 NSCLC specimens sections revealed that the positive expression of DKK1 is correlated with lymph nodes metastasis; it might be a predictor of cancer metastasis. With respect to prognosis, positive expression of DKK1 protein correlated with a dismal 5-year survival. DKK1 is upregulated in NSCLC tissues compared with the matched normal tissues. So it is possible that DKK1 is involved in the progression of NSCLC.

DKK1 is a secreted protein that contains a signal peptide sequence and two cysteine-rich domains and functions as a negative regulator of the Wnt signaling [6,7]. In addition, DKK1 was reported to be a downstream target of β-catenin/T-cell factor and participates in a negative feedback loop in the Wnt signaling in colon cancer cells [24,25]. Studies have indicated that overexpression of DKK1 was associated with poor prognosis [14,19,21], while little is known about the function of DKK1 in NSCLC. To further study the biological effects of DKK1 in ivtro, firstly, we verified that overexpression of DKK1 promotes migration and invasion in human NSCLC cell line 95C. Meanwhile, the overexpression of DKK1 upregulated the expression of metastasis related proteins, but the detailed mechanism need to be further study. Furthermore, using eukaryotic expression of DKK1 shRNA, we showed that knockdown of DKK1 suppresses the migration and invasion of NSCLC cell line LTEP-a-2.The results indicate that DKK1 might have a positive role in the progression of NSCLC, but the mechanism involved in invasion needs further studying. 

However, some studies have reported that DKK1 suppresses cell growth and migration [16,26]which suggests that there might be other roles of DKK1 in the Wnt/β-catenin signaling. DKK1 might play diverse biological roles in different type of cancer cells. A retrospective analysis of DKK1 expression showed that DKK1 was altered during PCa progression [27]. So far, only a few studies have been reported regarding the roles of DKK1 in NSCLC, mainly focusing the diagnosis and prognosis values [12,21,28-30]. The role of DKK1 in NSCLC, its mechanism of function and clinical aspects need further studies. 

In summary, although the detailed function of DKK1 in NSCLC progression is not well clarified, our results suggest the potential roles of DKK1 in the promotion of migration and invasive growth in NSCLC cell lines, and that it could serve as a novel therapeutic target for NSCLC.

## Supporting Information

Figure S1 **Disease-free survival curve classified according to DKK1 expression for all patients plotted by Kaplan–Meier methods.**
(TIF)

Table S1 **Primers sequence used for real-time PCR.**
(DOC)

Table S2 **Differencial expression of genes in 95C transfected with Pcmv-Tag2b-DKK1 relative to 95C transfected with Pcmv-Tag2b.**
(DOC)
