# Supplementary figures and images for: Dickkopf-1 Is Oncogenic and Involved in Invasive Growth in Non Small Cell Lung Cancer
Source: PLoS One. 2013 Dec 31;8(12):e84944. doi: 10.1371/journal.pone.0084944 (PMC3877398; doi:10.1371/journal.pone.0084944)

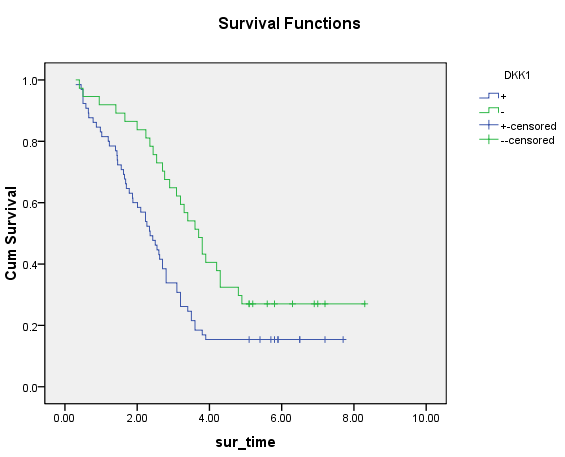

Supplement: Figure S1 — Disease-free survival curve classified according to DKK1 expression for all patients plotted by Kaplan–Meier methods. (TIF) [file pone.0084944.s001.tif]
