# Supplementary material for: Dickkopf-1 Is Oncogenic and Involved in Invasive Growth in Non Small Cell Lung Cancer
Source: PLoS One. 2013 Dec 31;8(12):e84944. doi: 10.1371/journal.pone.0084944 (PMC3877398; doi:10.1371/journal.pone.0084944)
Supplement: Table S1 — Primers sequence used for real-time PCR. (DOC) [file pone.0084944.s002.doc]

**Table S1** Primers sequence used for real-time PCR

| **Gene name** | **Primer sequence(5'-3')** | **product（bp)** |
| --- | --- | --- |
| **Cyclin D1** | ACGAAGGTCTGCGCGTGTT | 323 |
|  | CCGCTGGCCATGAACTACCT |  |
| **Bcl-2** | TCCGCATCAGGAAGGCTAGA | 113 |
|  | AGGACCAGGCCTCCAAGCT |  |
| **BAX** | GGGTGGTTGGGTGAGACTC | 199 |
|  | AGACACGTAAGGAAAACGCATTA |  |
| **Akt-1** | GCACAAACGAGGGGAGTACAT | 113 |
|  | CCTCACGTTGGTCCACATC |  |
| **MMP2** | GGCCCTGTCACTCCTGAGAT | 474 |
|  | GGCATCCAGGTTATCGGGGA |  |
| **VEGFC** | AGGAGGGCAGAATCATCACG | 405 |
|  | TATGTGCTGGCCTFGGTGAG |  |
