# Supplementary material for: Dickkopf-1 Is Oncogenic and Involved in Invasive Growth in Non Small Cell Lung Cancer
Source: PLoS One. 2013 Dec 31;8(12):e84944. doi: 10.1371/journal.pone.0084944 (PMC3877398; doi:10.1371/journal.pone.0084944)
Supplement: Table S2 — Differencial expression of genes in 95C transfected with Pcmv-Tag2b-DKK1 relative to 95C transfected with Pcmv-Tag2b. (DOC) [file pone.0084944.s003.doc]

**Table S2** Differencial expression of genes in 95C transfected with Pcmv-Tag2b-DKK1 relative to 95C transfected with Pcmv-Tag2b

| **Gene name** | **Relative fuction** | **Fold change** | ***P* value** |
| --- | --- | --- | --- |
| **Cyclin D1** | cell cycle | 1.21 | 2.12E-02 |
| **Bcl-2** | apoptosis | -1.07 | 7.90E-03 |
| **BAX** | apoptosis | 1.35 | 4.32E-02 |
| **Akt-1** | signaling pathway | -1.49 | 3.17E-04 |
| **MMP2** | invasion and metastasis | 7.78 | 4.83E-03 |
| **VEGFC** | invasion and metastasis | 2.13 | 1.51E-02 |
